# Child Abuse and Neglect and Obsessive–Compulsive Personality Traits: Effects of Attachment, Intolerance of Uncertainty, and Metacognition

**DOI:** 10.1007/s40653-024-00644-3

**Published:** 2024-07-09

**Authors:** Emily Gray, Naomi Sweller, Simon Boag

**Affiliations:** School of Psychological Sciences, Macquarie University, Australian Hearing Hub, 16 University Avenue, Sydney, NSW 2109 Australia

**Keywords:** Child abuse and neglect, Mediation, Metacognition, Intolerance of uncertainty, Obsessive–compulsive

## Abstract

Child Abuse and Neglect (CAN) is extensively implicated as a risk factor preceding the development of Obsessive–Compulsive Personality Traits (OCPT). Nevertheless, the majority of individuals with a history of CAN do not go on to develop OCPT. To date, little research has investigated potential model networks that may help contribute to explaining why CAN sometimes leads to OCPT and not at other times. Thus, this study aimed to investigate whether attachment-anxiety, intolerance of uncertainty, and metacognition have indirect effects in the association between CAN and OCPT in various network models. Undergraduate psychology students (*N* = 291) participated in an anonymous 30-min online survey consisting of a series of self-report questionnaires regarding child abuse and neglect, attachment, intolerance of uncertainty, metacognition, OCPT, and depression. Bootstrapped serial mediation revealed attachment-anxiety and intolerance of uncertainty had a serial-mediation effect in the association between CAN and OCPT. Serial mediation was not found for metacognition and attachment-anxiety. However, metacognition alone mediated between child emotional abuse and OCPT. These findings expand our currently limited knowledge regarding the etiology of OCPT and suggest that attachment-anxiety, intolerance of uncertainty, and metacognition may be important contributors for understanding the development of OCPT following CAN exposure. The potential clinical utility for both assessment and treatment are discussed.

Child abuse and neglect has a global prevalence of approximately 30% (Moody et al., 2018) and a dose-dependent association with numerous poor mental and physical health, and social and behavioural outcomes (Hughes et al., 2017; Petruccelli et al., 2019). *Child abuse and neglect* (CAN) involves exposure before 18-years of age to caregiver-perpetrated abuse (physical, sexual and emotional) and neglect (physical and emotional; Boullier & Blair, 2018). A plethora of studies identify CAN as risk factors in the development of sub-clinical and clinical *Obsessive–Compulsive Personality Traits* (OCPT; Afifi et al., 2011; Battle et al., 2004; Bernstein et al., 1998; Grover et al., 2007; Hengartner et al., 2013; Lobbestael et al., 2010; Roberts et al., 2008; Tyrka et al., 2009). OCPT encompass Obsessive–Compulsive Personality Disorder (OCPD)-related characteristics including perfectionism, overconscientious, absolute devotion to productivity, excessive control over one’s internal and external environment, reluctancy to delegate, moral scrupulosity, preoccupation with order and details, rigidity, and interpersonal and affective difficulty (American Psychiatric Association, 2022). Obsessive–compulsive personality can be considered along a continuum or set of continua whereby maladaptive impulse control forms one pole and maladaptive overcontrol constitutes the other, with ‘undisturbed’ existing somewhere in between (Samuel et al., 2012; Trull & Widiger, 2013). While both poles are potentially problematic, obsessive–compulsive personality typically belongs along the overcontrol pole-end. While OCPD represents the clinical diagnosis, OCPT are present in sub-clinical populations and are linked with suicide-related outcomes, reduced quality of life, and moderate psychosocial, interpersonal, and occupational impairment (Bowen et al., 2019; Morreale et al., 2021; Soeteman et al., 2008). Despite this, OCPT aetiological research generally remains both indeterminate and limited (Diedrich & Voderholzer, 2015).

There is evidence for a positive association between CAN and adult obsessive–compulsive pathology. Several clinical studies report that OCPD is positively associated with both child sexual and emotional abuse (Battle et al., 2004; Bernstein et al., 1998; Johnson et al., 2003; Lobbestael et al., 2010; Roberts et al., 2008). At the same time, self-reported CAN are positively associated with sub-clinical OCPT after controlling for depression (Afifi et al., 2011; Grover et al., 2007; Hengartner et al., 2013; Tyrka et al., 2009). In contrast, Johnson et al. (1999) found no association between CAN and OCPT, however, depression was neither excluded nor controlled for. As depression distorts reports of OCPT due to OCPT psychopathology closely approximating the distress of mood disorders (Case et al., 2007), controlling for depression is crucial when assessing CAN and OCPT.

## Potential Mediators of CAN-OCPT

Nevertheless, the majority of individuals that experience child abuse and neglect do not develop OCPT in adulthood (Gunay-Oge et al., 2020). Consequently, there may be mediating variables that help account for this development of OCPT following CAN exposure. Potential candidates include metacognition (Rees & Anderson, 2013) and intolerance of uncertainty (Wheaton & Ward, 2020) which are each associated with CAN and OCPT independently, and attachment which mediates between CAN and OCPT (Gray & Boag, in press). To our knowledge, no study has yet investigated whether these factors together may form various model networks that may help explain why CAN, at times, leads to OCPT and not at other times. This led the present study to investigate whether attachment, intolerance of uncertainty, and metacognition may form various model networks in the association between child abuse and neglect and OCPT.

## Attachment

A factor identified as contributing to the association between child abuse and neglect and OCPT is *attachment* (Gray & Boag, in press). Attachment is conceptualised as an individual’s characteristic manner of receiving intimacy from and bonding with significant others (Levy et al., 2011). Attachment develops from internal working models of self and of others which are cognitive and affective mental models concerning self-evaluation, and the dependability and accessibility of others, respectively (Ainsworth & Bowlby, 1991; Sherry et al., 2007). The psychological foundation of these internal working models is one’s interactions with primary caregiver(s) (Ainsworth & Bowlby, 1991): namely, the quality of emotional responses provided, and the perceived feedback from these interactions (Bizzi et al., 2015). Internal working models largely act outside of consciousness, assimilating social information in a consistent and confirmatory manner (Campbell & Marshall, 2011). Thus, an individual’s beliefs and behaviours regarding their worthiness (i.e., self-image) and their acceptability to attachment figures are informed by internal working models across the lifespan (Anders & Tucker, 2000). This temporal continuity means the attachment developed during childhood remains relatively stable and forms the model for later relationships (Sutton, 2019).

Caregiver-perpetrated abuse and neglect are times of distress whereby caregivers cannot be relied upon. If there is no support from an alternative significant other(s), then the development of secure internal working models is disrupted (Ainsworth & Bowlby, 1991; Kowalski et al., 2023; Lin et al., 2020; Luby et al., 2019; McEwen & McEwen, 2017; Wan et al., 2019). In response, internal working models depicting the self as worthless and others as both unavailable and rejecting may form and underly the development of an insecure attachment (Ainsworth & Bowlby, 1991; Baer & Martinez, 2006; Cyr et al., 2010; Widom et al., 2018; Yumbul et al., 2010). Insecure attachment typically develops along the continuums of attachment-anxiety (i.e., ‘internal working model of the self’ dimension) and attachment-avoidance (i.e., ‘internal working model of others’ dimension; Ainsworth & Bowlby, 1991; Lin et al., 2020). While the literature sometimes combines these dimensions to form various discrete categories (Bartholomew & Horowitz, 1991), a dimensional approach provides greater reliability, validity, and statistical power (Fraley et al., 2000; Griffin & Bartholomew, 1994) and is the favoured approach.

Insecure attachment is an important factor associated with both CAN and OCPT. Child emotional, physical, and sexual abuse are all positively associated with adult attachment insecurity within both student and community samples (Cyr et al., 2010; Erozkan, 2016; Lin et al., 2020; Yumbul et al., 2010). Moreover, Yumbul et al. (2010) found that insecurely attached adults reported significantly more abuse and neglect than controls. Although the cross-sectional nature of the aforementioned studies prevents establishing directionality, Carlson’s (1998) prospective longitudinal finding that insecure attachment at 19 years was related to infant abuse and neglect suggests CAN precedes development of attachment insecurity. Internal working models underlying insecure attachment are also associated with OCPT. Namely, self-reported insecure attachment within undergraduates positively associates with OCPT (Brennan & Shaver, 1998; Zakiei et al., 2017). Similarly, in comparison to controls, patients with OCPD report significantly greater fearful attachment (Wiltgen et al., 2015). Thus, attachment may plausibly mediate between CAN and OCPT.

In line with this, Gray and Boag (in press) recently found that attachment-anxiety appears to mediate between child abuse and neglect and OCPT. As is well documented, exposure to the adverse, poor-quality caregiving typical of CAN environments likely gives rise to attachment-anxiety (Cyr et al., 2010; Lin et al., 2020; Widom et al., 2018). Attachment-anxiety is underpinned by a model of the self as having a perceived inability to cope with threat and anguishing rejection and abandonment, and, therefore, needing absolute internal and external control (i.e., perfectionism and rigidity; Hertler, 2014; Lin et al., 2020; Yumbul et al., 2010). In turn, attachment-anxiety may increase OCPT-like characteristics and interpersonal problems as a means to gain this control (Clark et al., 2020). While these behaviours act as a coping mechanism during childhood, they precipitate maladaptive adult OCPT (Lyddon & Sherry, 2001). As developmental psychopathology has multifactorial determinants, attachment-anxiety is likely not sufficient for explaining OCPT and is thus a possible candidate for a broader model network that contributes to explaining why CAN, at times, leads to OCPT and not at other times.

## Intolerance of Uncertainty

A second factor that may be relevant in a model network that helps explain the association between child abuse and neglect and OCPT is *intolerance of uncertainty*. Intolerance of uncertainty is a cognitive dispositional bias involving negative beliefs about uncertainty and the ability to cope with distress from ambiguity (Buhr & Dugas, 2006; Dugas et al., 2004). Intolerance of uncertainty influences the interpretation, perception, and response to uncertainty at a cognitive, emotional, and behavioural level (Buhr & Dugas, 2006; Clark et al., 2020). Individuals high in intolerance of uncertainty have an incapacity to tolerate ambiguity and an absence of sufficient information, frequently engaging in maladaptive certainty-seeking behaviours (Carleton et al., 2012). As intolerance of uncertainty forms during psychological and emotional development, it is influenced by childhood familial and caregiving experiences (Sanchez et al., 2016). Indeed, intolerance of uncertainty may develop through exposure to caregiver-perpetrated child abuse and neglect whereby this adverse caretaking instils a sense of incompetency and inadequacy (Wright et al., 2017). This situation closely reflects those beliefs associated with attachment-anxiety in which there is a lack of self-worth that manifests as a fear (i.e., uncertainty) of rejection and ability to cope with threat (Wright et al., 2017).

Reflecting this, attachment-anxiety has been identified as a factor in the development of intolerance of uncertainty. Recent cross-sectional mediation studies suggest that self-reported attachment-anxiety is positively associated with self-reported intolerance of uncertainty, and this intolerance of uncertainty mediates between attachment-anxiety and worry (Clark et al., 2020; Wright et al., 2017). A longitudinal examination found that insecure attachment (relative to secure attachment) at 6-years of age was related to greater adulthood intolerance of uncertainty 15 years later, suggesting attachment-anxiety precedes development of intolerance of uncertainty (Zdebik et al., 2018). Within an attachment theory perspective, the attachment-anxiety orientation discussed above leads to the hyperactivation of the attachment system. This hyperactivation of the attachment system may predispose one to internalise uncertainty as dangerous and indicative of threat (Wright et al., 2017). This situation further hyperactivates the attachment system, driving the use of certainty-seeking behaviours, thereby instilling a subjective perception of certainty (Clark et al., 2020).

Intolerance of uncertainty is a transdiagnostic construct linked with several psychopathologies, indicating diverse manifestations of uncertainty-reducing behaviours (Shihata et al., 2016; Wheaton & Ward, 2020). Uncertainty-reducing behaviours are generally defined as behaviours employed with the aim of reducing or avoiding perceived threat and associated distress, but which maintain psychopathology by preventing fear disconfirmation (Hebert & Dugas, 2019). OCPT may reflect one such manifestation of uncertainty-reducing behaviours. Early theoretical accounts related OCPT behaviours to difficulties experiencing uncertainty, change and the unexpected (Mallinger, 1984). Similarly, Pollak (1987) suggested that the need for excessive control over others and the environment was driven by a fear of facing the unknown. Empirically, intolerance of uncertainty was significantly associated with OCPT when using information-seeking during a cognitive test (a behavioural measure of reducing uncertainty; Gallagher et al., 2003). Although performance on Gallagher et al.’s (2003) behavioural measure may have been confounded by the preoccupation with detail and perfectionism aspects of OCPT, a recent self-report study found that individuals with OCPT had significantly greater intolerance of uncertainty compared to community controls, and that intolerance of uncertainty significantly predicted OCPT (Wheaton & Ward, 2020). Thus, behavioural manifestations of OCPT, which represent efforts to exert excessive control and achieve perfectionism in order to eliminate uncertainty, are likely uncertainty-reducing behaviours evolved in response to an inability to tolerate uncertainty (Wheaton & Ward, 2020).

**Fig. 1:**
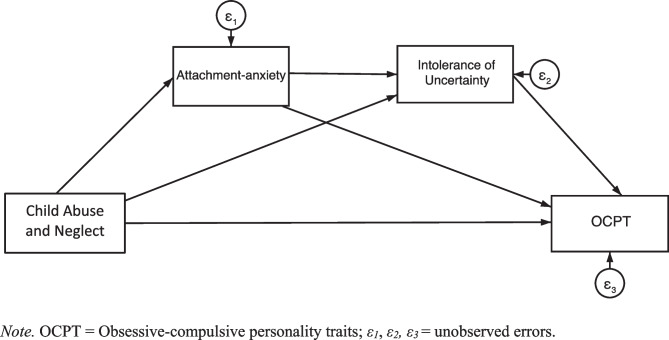
Model with Attachment-Anxiety and Intolerance of Uncertainty as Serial Mediators. *Note.* OCPT = Obsessive–compulsive personality traits; *ε*_*1*_, *ε*_*2*_*, ε*_*3*_ = unobserved errors

In summary, attachment-anxiety precedes and is associated with intolerance of uncertainty, and intolerance of uncertainty is associated with OCPT. Taken together with the finding that attachment-anxiety mediates between CAN and OCPT (discussed above), attachment-anxiety and intolerance of uncertainty may plausibly form a serial-mediation path between CAN and OCPT in a model network (see Fig. 1).

## Metacognition

A third potential mechanism that may contribute to explaining the association between child abuse and neglect and OCPT in a model network is *metacognition.* Metacognition, or knowledge about cognition, involves one’s beliefs regarding thought processes, along with strategies to appraise, control, and monitor thinking (Rees & Anderson, 2013). As metacognition has a prolonged development throughout childhood and adolescence, it is proposed that exposure to CAN likely develops maladaptive metacognitive beliefs about needing to use worry and threat monitoring to avoid emotional distress (Myers & Wells, 2015; Schneider, 2008; Weil et al., 2013). The influence of metacognition on psychopathology can be best understood by Wells’ (2000) Self-Regulatory Executive Function (S-REF) model. The core idea here is that psychopathology develops from a dysfunctional prolonged processing of otherwise typically transient negative emotional responses (e.g., anxiety or worthlessness). This prolonged processing is cognitive attentional syndrome, entailing rumination, attention directed on external and internal threat, and maladaptive coping strategies (Myers & Wells, 2015). Cognitive attentional syndrome arises from erroneous positive and/or negative metacognitive beliefs. Positive beliefs depict the advantages of the cognitive activities performed by cognitive attentional syndrome, while negative beliefs depict the danger of said activities and of thoughts (Wells, 2008). This cognitive attentional syndrome maintains and intensifies one’s negative self-perception and affective state resulting in a metacognitive inflexibility to implement alternate thought styles (Myers & Wells, 2015). Ultimately, this emotion regulation dysfunction descends into psychopathological states (e.g., anxiety; Wells, 2000).

Metacognition is an important factor associated with both CAN and obsessive–compulsive symptoms. For example, self-reported child *emotional* abuse was found to positively associate with both rumination (Raes & Hermans, 2008) and metacognitive beliefs as measured by the Metacognitions Questionnaire – 30 (Myers & Wells, 2015). Moreover, retrospectively reported child sexual and emotional abuse were developmental antecedents for rumination reported 30 months later (Spasojević & Alloy, 2002). Regarding obsessive–compulsive symptoms, prospective studies within student samples found that maladaptive metacognitive beliefs predicted obsessive–compulsive symptoms (Myers et al., 2009; Sica et al., 2007). From a neurocognitive perspective, undergraduates with pronounced OCPT displayed executive dysfunction in attentional shifting – a phenomenon referred to as obsessional slowness (García-Villamisar & Dattilo, 2015). Such slowness is proposed to result from preservation and rumination (García-Villamisar & Dattilo, 2015; Veale, 1993). Given the above, metacognition may plausibly mediate between CAN and OCPT.

**Fig. 2:**
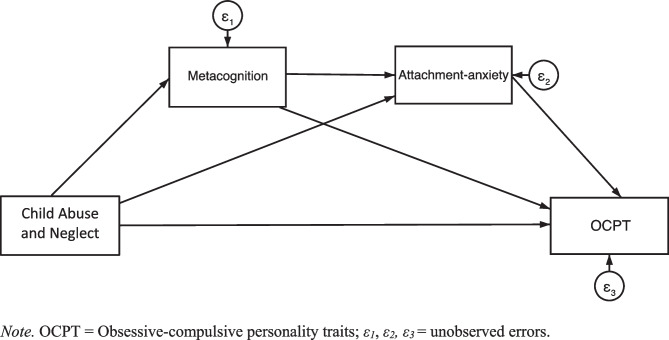
Model with Metacognition and Attachment-Anxiety as Serial Mediators. *Note.* OCPT = Obsessive–compulsive personality traits; *ε*_*1*_, *ε*_*2*_*, ε*_*3*_ = unobserved errors

Gray and Boag (in press) recently found, however, that metacognition did not appear to mediate the association between CAN and OCPT. Their finding may have been due to examining CAN as a general class, whereas past research suggests metacognition is associated with child *emotional* abuse specifically (Myers & Wells, 2015). It is reasonable to theorise then that metacognition may mediate between only child emotional abuse and OCPT. Moreover, Myers and Wells (2015) also suggest that metacognitive beliefs mediate between child emotional abuse and attachment-anxiety. Given attachment-anxiety mediates between CAN and OCPT (as discussed earlier), metacognition and attachment-anxiety may plausibly form a serial-mediation path between child emotional abuse and OCPT in a model network (see Fig. 2).

## Aim

To date, no study has integrated attachment, intolerance of uncertainty, and metacognition to examine whether these variables can help contribute to accounting for why CAN sometimes leads to OCPT and not at others. Noting that no single study can possibly address all mediating factors of any association, support for a postulated mediation merely means the results are consistent with that model *as well as* other possible models, whereas a failure to support provides a disconfirmation of the postulated causal associations (Kwok et al., 2019; Salthouse, 2011). Thus, we adopt here an informative approach by examining multiple postulated mediation models using the same data, in order to eliminate possible alternatives and thereby increase confidence in the target model/s (Salthouse, 2011). Hence the aim of the present study is to examine whether attachment-anxiety, intolerance of uncertainty, and metacognition form possible chain mechanisms between child abuse and neglect and OCPT by testing multiple competing serial mediation models.

## Hypotheses

All hypotheses are examined controlling for depression (Case et al., 2007). It is hypothesised that:

**H1:** Attachment-anxiety and intolerance of uncertainty significantly, positively serially mediates between CAN and OCPT.

**H2:** Metacognition and attachment-anxiety significantly, positively serially mediates between child emotional abuse and OCPT.

## Method

### Participants

Three hundred undergraduate psychology students completing research in exchange for course credit were recruited from an undergraduate participant pool system and self-selected into the study based on an advertisement in the participant pool. In order to identify careless responding, attention checks, which effectively identify careless responding and increase data quality (Shamon & Berning, 2020), were included in the present survey (“Please respond with strongly disagree”; “Please respond with false”; and “Please respond with agree slightly”). From the total sample, nine participants were excluded for failing more than one attention check based on the recommendation of Olatunji et al., (2007; Haidt et al., 1994) who identified during scale construction that careless responders failed the two attention checks. There was nothing unique about the excluded participants, nor were there any patterns across them.^1^ The sample size required for 80% power at the .05 significance level was estimated to be 200–300 participants based on Wolf et al.’s (2013) assessment of sample size requirements. Thus, the present study’s sample size of 291 was sufficient. Inclusion criteria required participants to have a fluent level of English and to be strictly 18-years of age or older. The sample was aged 18 to 61 (*M* = 20.6, *SD* = 6.1) and females constituted 82.8% (*n* = 241). Ethnicity was reported as 143 as White/Caucasian (49.1%), 60 as Asian (20.6%), 29 as South Asian (10%), 29 as Middle Eastern (10%), 2 as Black or African American (0.7%), 1 as Aboriginal or Torres Strait Islander (0.3%), and 27 as other (9.3%).

### Measures

#### Child Abuse and Trauma Scale (CATS)

The CATS (Sanders & Becker-Lausen, 1995) retrospectively measures the frequency of adverse child and adolescent experiences committed by caretakers. The CATS is a 38-item self-report scale with a total score and four subscales: a 7-item emotional abuse subscale (example item: “Did your parents insult you or call you names?” and “Did you feel disliked by either of your parents?”; Kent & Waller, 1998), a 14-item neglect subscale (example item: “As a child, did you feel unwanted or emotionally neglected?” and “As a child, did you have to take care of yourself before you were old enough?”), a 6-item sexual abuse subscale (example item: “Did your relationship with your parents ever involve a sexual experience?” and “Did you have traumatic sexual experiences as a child or teenager?”), and a 6-item physical abuse/punishment scale (example item: “Were you expected to follow a strict code of behaviour in your home?” and “Did your parents ever hit or beat you when you did not expect it?”). This study used only the total score and the emotional abuse subscale. Items are rated on a 5-point Likert scale ranging from 0 (*Never*) to 4 (*Always*), with higher average total and emotional abuse subscale scores (theoretically ranging from 0–4) indicative of greater CAN severity. The CATS has demonstrated good convergent validity through positive significant correlations with depression, dissociation, and interpersonal relationship impairment (Sanders & Becker-Lausen, 1995). Moreover, the mean CATS score for a sample with dissociative identity disorder, a disorder associated with an extremely high child abuse and neglect frequency, was statistically significantly four times that of an undergraduate sample (Sanders & Becker-Lausen, 1995). The positive, significant test–retest reliability of the CATS over a 6-to-8-week period (*r* = .71 to .91) demonstrates strong temporal stability (Sanders & Becker-Lausen, 1995). The mean and standard deviation for the CATS within the present sample was as expected and comparable to that generally found for this scale (see *Descriptive Statistics* section; Barlow et al., 2017; Briggs & Price, 2009; Hocking et al., 2016; Jenkins et al., 2013; Sanders & Becker-Lausen, 1995). The CATS total score and emotional abuse subscale score have demonstrated strong internal consistency (α = 0.90 and α = 0.88, respectively; Kent & Waller, 1998; Sanders & Becker-Lausen, 1995). The CATS total and emotional abuse subscale were both found to have excellent reliability in this study with a Cronbach’s alpha of 0.96 and 0.94, respectively.

#### Experiences in Close Relationships – Revised Questionnaire (ECR-R)

The ECR-R (Fraley et al., 2000), is a 36-item self-report questionnaire measuring underlying adult attachment patterns on two dimensions: attachment-avoidance (18-items; example item: “I prefer not to be too close to romantic partners” and “I don’t feel comfortable opening up to romantic partners”) and attachment-anxiety (18-items; example item: “I often worry that my partner will not stay with me” and “I'm afraid that I will lose my partner's love”). This study used only the attachment-anxiety scale. Items are rated on a 7-point Likert scale ranging from 1 (*strongly disagree*) to 7 (*strongly agree*), with higher average scores (theoretically ranging from 1–7) indicating greater attachment-anxiety. The ECR-R has demonstrated good convergent validity by significantly relating to diary ratings of attachment-anxiety-related emotions in romantic interactions (Sibley et al., 2005). The ECR-R is found to have good temporal stability through repeated measures over a 6-week period (Sibley & Liu, 2004). The ECR-R attachment-anxiety scale has demonstrated excellent internal consistency (α > 0.90; Sibley & Liu, 2004). The ECR-R attachment-anxiety scale was found to have excellent reliability in this study with a Cronbach’s alpha of 0.93.

#### Intolerance of Uncertainty Scale (IUS)

The IUS (Freeston et al., 1994) is a 27-item self-report questionnaire assessing the behavioural, emotional, and cognitive reactions to uncertainty and situations involving ambiguity, along with attempts at controlling the future (example item: “Uncertainty makes life intolerable” and “Unforeseen events upset me greatly”). Items are rated on a 5-point Likert scale ranging from 1 (*not at all characteristic of me*) to 5 (*entirely characteristic of me*), with higher sum total scores (theoretically ranging from 27–135) indicating greater intolerance of uncertainty. The IUS has displayed good convergent validity through positive significant correlations with worry, depression symptoms, and anxiety, and good temporal stability with a significant, positive test–retest reliability over a 5-week period (*r* = .74; Buhr & Dugas, 2002). The IUS has displayed excellent internal consistency (α = .94; Buhr & Dugas, 2002). The IUS was found to have excellent reliability in this study with a Cronbach’s alpha of 0.95.

#### The Metacognitions Questionnaire – 30 (MCQ-30)

The MCQ-30 (Wells & Cartwright-Hatton, 2004) is a 30-item self-report questionnaire that assesses individual differences in metacognitions associated with psychopathology (example item: “My worrying could make me go mad” and “Not being able to control my thoughts is a sign of weakness”). Items are rated on a 4-point Likert scale ranging from 1 (*do not agree*) to 4 (*agree very much*) with higher sum scores (theoretically ranging from 30–120) indicative of greater dysfunctional metacognitions. The MCQ-30 has demonstrated good convergent validity through positive, significant correlations with obsessive–compulsive symptoms, worry, and trait anxiety, and good temporal stability through a positive, significant test–retest reliability (*r* = .75; Wells & Cartwright-Hatton, 2004). The MCQ-30 has demonstrated excellent internal consistency (α = 0.93; Wells & Cartwright-Hatton, 2004). The MCQ-30 was found to have excellent reliability in this study with an identical Cronbach’s alpha of 0.93.

#### Outcome Measures

Two measures were selected for testing OCPT to assess differing conceptualisations of these traits as the literature remains unclear on the superiority of either a DSM criteria-based or maladaptive trait approach for the measurement of disordered personality (Widiger & Hines, 2022). As such, the SNAP-2 measures the OCPD DSM-5 criteria (American Psychiatric Association, 2013), while the FFOCI-SF is derived from maladaptive variants of the Five Factor Model of personality (Costa & McCrae, 1992). Given that there is still some debate as to how best conceptualise OCPT, including both measures will help assess a broader spectrum of OCPT compared to simply using one measure, while also providing grounds for assessing convergent validity.

##### Schedule for Non-adaptive and Adaptive Personality – 2 (SNAP-2)

The SNAP-2 (Clark, 2014) measures both healthy and pathological personality traits. This study used only the OCPD diagnostic scale (SNAP-2 OCPD), a 25-item self-report scale that dimensionally measures the DSM-5 (American Psychiatric Association, 2013) OCPD criteria (example item: “I never get so caught up in my work that I neglect my family and friends” and “I don’t consider a task finished until it is perfect”). Items are rated on a dichotomous 1 (*True*) or 0 (*False*) scale, with higher sum scores (theoretically ranging from 0–25) indicating greater OCPD symptomology. The SNAP-2 has displayed good convergent validity through positive, significant correlations with other self-report personality disorder questionnaires (Widiger & Boyd, 2009) and interview-based dimensional diagnoses (median *r* = .61; Clark, 2014). The SNAP-2 has also demonstrated adequate temporal stability over a two-year period with a positive, significant test–retest reliability (*r* = .61; Samuel et al., 2011). The SNAP-2 OCPD has demonstrated acceptable internal consistency (α = .79; Samuel et al., 2012). The SNAP-2 OCPD was found to have acceptable reliability in this study with a Cronbach’s alpha of 0.72.

##### Five Factor Obsessive–Compulsive Inventory – Short Form (FFOCI-SF)

The FFOCI-SF (Griffin et al., 2018) is a 48-item self-report questionnaire that assesses maladaptive traits associated with OCPD as derived from the Five-Factor Model of personality (example item: “I’m something of a perfectionist” and “I’m fanatical about getting things done when they need to be”). Items are rated on a 5-point Likert scale ranging from 1 (*strongly disagree*) to 5 (*strongly agree*), with higher sum scores (theoretically ranging from 48–240) indicating greater presence of OCPD traits. The FFOCI-SF is reported to have good convergent validity through positive, significant median correlations with the NEO-PI facets (*r* = .41 to .81) and established measures of OCPD (*r* = .34 to .55), including the SNAP-2 (*r* = .47; Samuel et al., 2012). The FFOCI-SF has also demonstrated acceptable-to-good internal consistency (*α* = .71 to .85; Samuel et al., 2012). The FFOCI-SF was found to have excellent reliability in this study with a Cronbach’s alpha of 0.91.

#### Covariate

##### International Personality Item Pool Depression Scale (IPIP-DEP)

The IPIP-DEP (Goldberg et al., 2006) is a 10-item self-report questionnaire (freely accessed online via https://ipip.ori.org/) that measures the tendency to experience negative affect and was used to control for depression (example item: “I am often down in the dumps” and “Feel that my life lacks direction”). Items are rated on a 5-point Likert scale ranging from 1 (*very inaccurate*) to 5 (*very accurate*), with higher sum scores (theoretically ranging from 10–50) indicative of greater negative affect. The IPIP-DEP is reported to have good convergent validity through a positive, significant correlation with the NEO-PI depressive subscale (Donnellan et al., 2006). The IPIP-DEP has also displayed good internal consistency (*α* = .88; Goldberg et al., 2006). The IPIP-DEP was found to have good reliability in this study with a Cronbach’s alpha of 0.86.

### Procedure

The present study was granted ethical approval from Macquarie University’s Human Research Ethics Committee (Ref: 520231262146197) and was pre-registered (AsPredicted #125162). Participants were blind to the study’s hypotheses and completed an anonymous 30-min Qualtrics survey as part of a larger project. After self-selecting into the study, participants received a link to complete the study at a location and time of their choosing. All participants provided consent to participate, and completed demographic questions regarding gender, age, and ethnicity. Participants then completed the *CATS, ECR-R, IUS, MCQ-30, IPIP-DEP, FFOCI-SF,* and *SNAP-2* where scale presentation and the order of items within each scale were randomised to control for order and priming effects. All participants were presented a debrief statement outlining the purpose and potential significance of this study.

### Design and Analysis

The present study comprised a non-experimental, cross-sectional design involving PROCESS macro modelling (Hayes, 2018). Preliminary data analyses were conducted using Stata/MP 17.0 software, and primary data analyses used IBM SPSS Statistics 27 software. Each proposed model was assessed twice: once with OCPT measured by the SNAP-2 (SNAP-2 OCPT) and once measured by the FFOCI-SF (FFOCI-SF OCPT). Depression was controlled for in all models. The proposed models are illustrated in Figs. 1 and 2.

For preliminary analyses, Spearman’s rank-order correlations were used to explore the bivariate relationships between the variables. Regarding primary data analyses, for the first and second hypothesis, model 6 of SPSS PROCESS macro was used with 5000 bootstrap resamples given the data were non-normally distributed (Hayes, 2018).

## Results

### Preliminary Analyses

#### Descriptive Statistics

For each model, examination of influence and distance revealed that 95% of the points were within plus or minus two standard deviations of the regression lines, suggesting no outliers within the present sample. Shapiro–Wilk tests revealed that depression, SNAP-2 OCPT and FOCCI-SF OCPT were normally distributed, whereas the CATS, attachment-anxiety, intolerance of uncertainty, and metacognition were non-normally distributed (Table 1). As such, nonparametric analyses were used for preliminary analyses and nonparametric bootstrapping for primary analyses. The assumption of independence of observations for these preliminary and primary analyses was met by the study design.

**Table 1:** Descriptive Statistics for Study Variables

Variable	*M*	*SD*	Min	Max	Skew	Kurtosis	Shapiro–wilk
Child Abuse and Trauma Scale	1.1	0.7	0.1	3.4	0.8	2.9	*p* < .001
Emotional Abuse CATS Subscale	1.5	1.0	0	4	0.6	2.5	*p* < .001
ECR-R Attachment-Anxiety	4.0	1.2	1.1	6.8	-0.2	2.5	*p* = .002
Intolerance of Uncertainty Scale	78.0	22.7	28	131	-0.1	2.4	*p* = .043
Metacognitions Questionnaire – 30	69.6	17.2	32	117	0.3	2.6	*p* = .033
FFOCI-SF	145.3	22.0	66	202	-0.3	3.3	*p* = .150
SNAP-2	13.6	4.3	2	24	-0.04	2.6	*p* = .850
IPIP-DEP	29.2	8.2	10	49	-0.1	2.5	*p* = .226

N = 291. *CATS* Child Abuse and Trauma Scale, *ECR-R* Experiences in Close Relationships – Revised, *FFOCI-SF* Five Factor Obsessive–Compulsive Inventory – Short Form, *SNAP-2* Schedule for Nonadaptive and Adaptive Personality – 2, *IPIP-DEP* International Personality Item Pool Depression Scale

The CATS, the primary predictor for all models, had adequate central tendency and variability (Table 1). That is, the mean frequency (and standard deviation) of child abuse and trauma events for the present sample was as expected, being comparable to that reported in the literature within small and large undergraduate samples (*M* = 0.7–0.9, *SD* = 0.4–0.6; Barlow et al., 2017; Hocking et al., 2016; Jenkins et al., 2013; Sanders & Becker-Lausen, 1995) and within non-clinical community samples (*M* = 1.2, SD = 0.7; Briggs & Price, 2009).

#### Bivariate Correlations

Spearman’s rank-order correlations were conducted to examine the bivariate relationships between the variables given some were not normally distributed (see Table 1). Cohen’s (1988) convention was utilised to determine the effect size of the coefficients (weak: *r*_*s*_ =| .1 -. 29 |; moderate: *r*_*s*_ =| .3—.49 |; strong: *r*_*s*_ ≥| .5 |). Displayed in Table 2, all the correlations were as expected except attachment-anxiety scores were not significantly correlated with SNAP-2 OCPT scores.

**Table 2:** Spearman’s Rank-Order Correlations Between the Study Variables

Variable	1	2	3	4	5	6
1. Child Abuse and Trauma Scale						
2. ECR-R Attachment-Anxiety	.35***					
3. Intolerance of Uncertainty Scale	.35***	.46***				
4. Metacognitions Questionnaire – 30	.41***	.45***	.69***			
5. FFOCI-SF	.18**	.18***	.48***	.42***		
6. SNAP-2	.25***	.11	.39***	.41***	.64***	
7. IPIP-DEP	.46***	.50***	.54***	.57***	.19***	25***

N = 291. *ECR-R* Experiences in Close Relationships –Revised, *FFOCI-SF* Five Factor Obsessive–Compulsive Inventory – Short Form, *SNAP-2* Schedule for Nonadaptive and Adaptive Personality – 2, *IPIP-DEP* International Personality Item Pool Depression Scale

**p < .01; ***p < .001

Convergent validity of the two OCPT scales was exhibited through a significant positive, strong correlation between SNAP-2 scores and FFOCI-SF scores (see Table 2). In relation to hypothesis 2, child emotional abuse CATS subscale scores had significant positive, moderate correlations with metacognition scores *r*_*s*_ = .42, *p* < .001 and attachment-anxiety scores *r*_*s*_ = .37, *p* < .001, and significant positive, weak correlations with SNAP-2 OCPT scores *r*_*s*_ = .24, *p* < .001, and FOCCI-SF OCPT scores *r*_*s*_ = .20, *p* < .001.

### Primary Analyses

#### Hypothesis 1

**Fig. 3:**
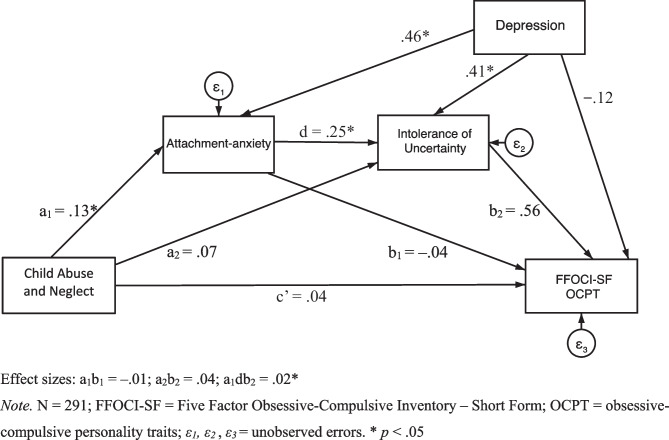
Model with Attachment-anxiety and Intolerance of Uncertainty as Mediators and FFOCI-SF as the Outcome. Effect sizes: a_1_b_1_ = –.01; a_2_b_2_ = .04; a_1_db_2_ = .02*. *Note.* N = 291; FFOCI-SF = Five Factor Obsessive–Compulsive Inventory – Short Form; OCPT = obsessive–compulsive personality traits; *ε*_*1*_*, ε*_*2*_, *ε*_*3*_ = unobserved errors. * *p* < .05

Regarding the first hypothesis, that attachment-anxiety and intolerance of uncertainty significantly, positively serially mediate between CAN and OCPT, Table 3 presents the serial mediation results. The results (Table 3) demonstrated that the standardised indirect effect of CAN on FFOCI-SF OCPT through both attachment-anxiety and intolerance of uncertainty was significant and positive in a sequential manner, demonstrated by bootstrapped confidence intervals that do not contain zero. The standardised indirect effect of CAN on FFOCI-SF OCPT through attachment-anxiety was non-significant, and the standardised indirect effect of CAN on FFOCI-SF OCPT through intolerance of uncertainty was also non-significant. The standardised direct effect of CAN on FFOCI-SF OCPT was non-significant. Thus, attachment-anxiety and intolerance of uncertainty appear to serially mediate between CAN and FFOCI-SF OCPT. Figure 3 displays the serial mediation model with standardised regression coefficients.

**Table 3:** Direct and Indirect Effects for the Structural Model

Model Pathway	*β*	Bootstrapped *SE*	Bootstrapped 95% CI	
			LL	UL
**Direct Effects**				
CAN → FFOCI-SF OCPT	.04	.06	–.077	.154
CAN → Attachment-anxiety	.13	.06	.006	.249
CAN → Intolerance of Uncertainty	.07	.05	–.027	.167
AA → IU	.25	.05	.142	.356
AA → FFOCI-SF OCPT	–.04	.07	–.190	.104
IU → FFOCI-SF OCPT	.56	.07	–.428	.685
**Indirect Effects**				
CAN → AA → FFOCI-SF OCPT	–.01	.01	–.029	.015
CAN → IU → FFOCI-SF OCPT	.04	.03	–.015	.095
CAN → AA → IU → FFOCI-SF OCPT	.02	.01	.001	.037

Statistical significance of all the direct and indirect paths remains the same when additionally controlling for age, gender, and ethnicity

N = 291. Controlling for Depression. *CAN* child abuse and neglect, *FFOCI-SF* Five Factor Obsessive–Compulsive Inventory – Short Form, *OCPT* obsessive–compulsive personality traits, *AA* attachment-anxiety, *IU* intolerance of uncertainty

**Fig. 4:**
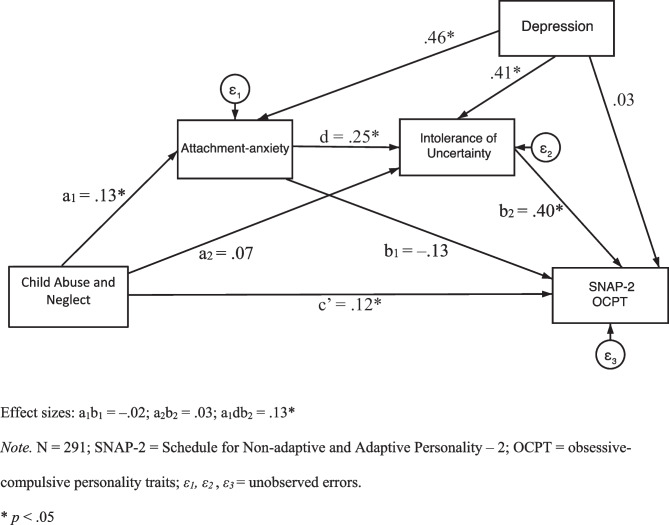
Model with Attachment-anxiety and Intolerance of Uncertainty as Mediators and SNAP-2 as the Outcome. Effect sizes: a_1_b_1_ = –.02; a_2_b_2_ = .03; a_1_db_2_ = .13*. *Note.* N = 291; SNAP-2 = Schedule for Non-adaptive and Adaptive Personality – 2; OCPT = obsessive–compulsive personality traits; *ε*_*1*_*, ε*_*2*_, *ε*_*3*_ = unobserved errors. * *p* < .05

Also, regarding the first hypothesis but with OCPT as measured by the SNAP-2, Fig. 4 displays the serial mediation model with standardised regression coefficients. The serial mediation results (see Table 4) demonstrated that the standardised indirect effect of CAN on SNAP-2 OCPT through both attachment-anxiety and intolerance of uncertainty was also significant and positive in a sequential manner.

**Table 4:** Direct and Indirect Effects for the Structural Model

Model Pathway	*β*	Bootstrapped *SE*	Bootstrapped 95% CI	
			LL	UL
**Direct Effects**				
CAN → SNAP-2 OCPT	.12	.06	.001	.242
CAN → Attachment-anxiety	.13	.06	.003	.246
CAN → Intolerance of Uncertainty	.07	.05	–.029	.168
AA → IU	.25	.05	.145	.358
AA → SNAP-2 OCPT	–.13	.07	–.260	.021
IU → SNAP-2 OCPT	.40	.06	.278	.525
**Indirect Effects**				
CAN → AA → SNAP-2 OCPT	–.02	.01	–.043	.004
CAN → IU → SNAP-2 OCPT	.03	.02	–.012	.070
CAN → AA → IU → SNAP-2 OCPT	.13	.01	.001	.027

Statistical significance of all the direct and indirect paths remains the same when additionally controlling for age, gender, and ethnicity

N = 291. Controlling for Depression. *CAN* child abuse and neglect, *SNAP-2* Schedule for Non-adaptive and Adaptive Personality – 2, *OCPT* obsessive–compulsive personality traits, *AA* attachment-anxiety, *IU* intolerance of uncertainty

In addition, the standardised indirect effect of CAN on SNAP-2 OCPT through attachment-anxiety was non-significant, and the standardised indirect effect of CAN on SNAP-2 OCPT through intolerance of uncertainty was also non-significant. The standardised direct effect of CAN on SNAP-2 OCPT was significant and positive. Thus, attachment-anxiety and intolerance of uncertainty appear to partially serially mediate between CAN and SNAP-2 OCPT.

#### Hypothesis 2

**Fig. 5:**
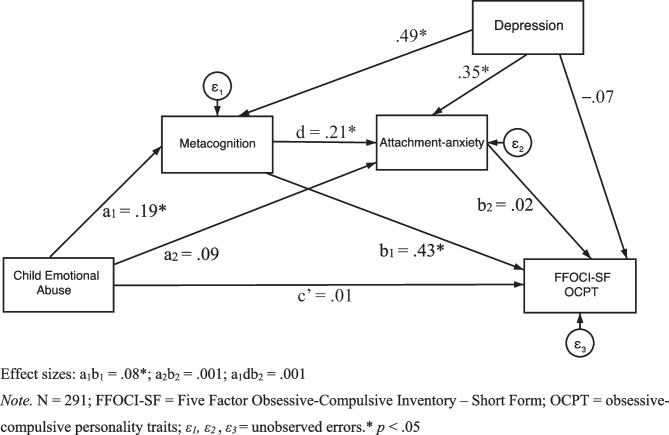
Model with Metacognition and Attachment-anxiety as Mediators and FFOCI-SF as the Outcome. Effect sizes: a_1_b_1_ = .08*; a_2_b_2_ = .001; a_1_db_2_ = .001. *Note.* N = 291; FFOCI-SF = Five Factor Obsessive–Compulsive Inventory – Short Form; OCPT = obsessive–compulsive personality traits; *ε*_*1*_*, ε*_*2*_, *ε*_*3*_ = unobserved errors.* *p* < .05

Regarding the second hypothesis that metacognition and attachment-anxiety significantly, positively serially mediate between child emotional abuse and OCPT, Fig. 5 displays the serial mediation model with standardised regression coefficients. The serial mediation results (see Table 5) demonstrated that the standardised indirect effect of child emotional abuse on FFOCI-SF OCPT through both metacognition and attachment-anxiety was non-significant in a sequential manner. In addition, the standardised indirect effect of child emotional abuse on FFOCI-SF OCPT through attachment-anxiety was also non-significant. However, the standardised indirect effect of child emotional abuse on FFOCI-SF OCPT through metacognition was significant and positive. The standardised direct effect of child emotional abuse on FFOCI-SF OCPT was non-significant.

**Table 5:** Direct and Indirect Effects for the Structural Model

Model Pathway	*β*	Bootstrapped *SE*	Bootstrapped 95% CI	
			LL	UL
**Direct Effects**				
CEA → FFOCI-SF OCPT	.01	.06	–.118	.128
CEA → Metacognition	.19	.05	.085	.295
CEA → Attachment-anxiety	.09	.06	–.040	.209
Metacognition → AA	.21	.07	.081	.337
Metacognition → FFOCI-SF OCPT	.43	.07	.302	.567
AA → FFOCI-SF OCPT	.02	.08	–.132	.168
**Indirect Effects**				
CEA → Mcog → FFOCI-SF OCPT	.08	.03	.034	.137
CEA → AA → FFOCI-SF OCPT	.001	.01	–.016	.021
CEA → Mcog → IU → FFOCI-SF OCPT	.001	.003	–.006	.008

Statistical significance of all the direct and indirect paths remains the same when additionally controlling for age, gender, and ethnicity

N = 291. Controlling for Depression. *CEA* child emotional abuse, *FFOCI-SF* Five Factor Obsessive–Compulsive Inventory – Short Form, *OCPT* obsessive–compulsive personality traits, *AA* attachment-anxiety, *Mcog* metacognition

**Fig. 6:**
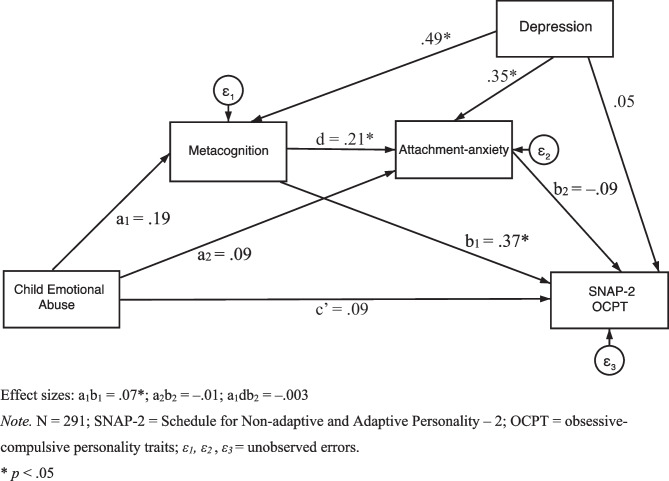
Model with Metacognition and Attachment-anxiety as Mediators and SNAP-2 as the Outcome. Effect sizes: a_1_b_1_ = .07*; a_2_b_2_ = –.01; a_1_db_2_ = –.003. *Note.* N = 291; SNAP-2 = Schedule for Non-adaptive and Adaptive Personality – 2; OCPT = obsessive–compulsive personality traits; *ε*_*1*_*, ε*_*2*_, *ε*_*3*_ = unobserved errors. * *p* < .05

Also, regarding the second hypothesis but with OCPT as measured by the SNAP-2, Fig. 6 displays the serial mediation model with standardised regression coefficients. The serial mediation results (see Table 6) demonstrated that the standardised indirect effect of child emotional abuse on SNAP-2 OCPT through both metacognition and attachment-anxiety was non-significant in a sequential manner. In addition, the standardised indirect effect of child emotional abuse on SNAP-2 OCPT through attachment-anxiety was also non-significant. However, the standardised indirect effect of child emotional abuse on SNAP-2 OCPT through metacognition was significant and positive (see Table 6). Furthermore, the standardised direct effect of child emotional abuse on SNAP-2 OCPT was non-significant.

**Table 6:** Direct and Indirect Effects for the Structural Model

Model Pathway	*β*	Bootstrapped *SE*	Bootstrapped 95% CI	
			LL	UL
**Direct Effects**				
CEA → SNAP-2 OCPT	.09	.06	–.034	212
CEA → Metacognition	.19	.05	–.084	291
CEA → Attachment-anxiety	.09	.06	–.036	.212
Metacognition → AA	.21	.06	.094	.331
Metacognition → SNAP-2 OCPT	.37	.06	.243	.488
AA → SNAP-2 OCPT	–.09	.07	–.226	.045
**Indirect Effects**				
CEA → Mcog → SNAP-2 OCPT	.07	.02	.029	.116
CEA → AA → SNAP-2 OCPT	–.01	.01	–.030	.007
CEA → Mcog → IU → SNAP-2 OCPT	–.003	.003	–.011	.002

Statistical significance of all the direct and indirect paths remains the same when additionally controlling for age, gender, and ethnicity

N = 291. Controlling for Depression. *CEA* child emotional abuse, *SNAP-2* Schedule for Non-adaptive and Adaptive Personality – 2, *OCPT* obsessive–compulsive personality traits, *AA* attachment-anxiety, *Mcog* metacognition

Thus, metacognition and attachment-anxiety do not appear to serially mediate between child emotional abuse and OCPT for both the FFOCI-SF and SNAP-2. However, metacognition does appear to mediate between child emotional abuse and OCPT, for both the FFOCI-SF and SNAP-2, controlling for depression.

## Discussion

The present study, to our knowledge, is the first to investigate whether attachment-anxiety, intolerance of uncertainty, and metacognition help contribute to explaining the relationship between CAN and OCPT.

### Hypothesis 1

Our first hypothesis, that attachment-anxiety and intolerance of uncertainty would significantly, positively serially mediate between CAN and OCPT controlling for depression, was supported for both measures of OCPT. In line with this prediction, the results demonstrated that attachment-anxiety and intolerance of uncertainty had a serial mediation effect: higher child abuse and neglect frequency predicted greater attachment-anxiety, which, in turn, predicted higher intolerance of uncertainty, which, in turn, predicted greater OCPT. Although causal conclusions cannot be drawn due to our study’s cross-sectional design, these results provide a preliminary step to assessing theoretical chain models and are consistent with proposed theoretical mechanisms (Clark et al., 2020; Hayes, 2018; Salthouse, 2011). Additionally, this preliminary finding extends existing cross-sectional and longitudinal work which similarly reports a temporal link between CAN and greater attachment-anxiety/insecure attachment (Carlson, 1998; Erozkan, 2016; Lin et al., 2020; Yumbul et al., 2010), and between greater attachment-anxiety and greater intolerance of uncertainty (Clark et al., 2020; Wright et al., 2017; Zdebik et al., 2018). Moreover, this finding extends prior cross-sectional research where greater intolerance of uncertainty is positively associated with greater OCPT (Gallagher et al., 2003; Wheaton & Ward, 2020). This finding further compliments and builds upon Gray and Boag’s (in press) recent work which implicated attachment-anxiety as one mechanism underlying the CAN***–***OCPT relationship. In line with developmental psychopathology expectations, the present study further suggests that attachment-anxiety *and* intolerance of uncertainty provide a chain mechanism that may help account for the development of OCPT following CAN.

In the context of attachment-theory, exposure to adverse, poor-quality caregiving typical of caregiver-perpetrated abusive or neglectful environments likely disrupts adaptive internal working model formation, consequently inducing attachment-anxiety (Lin et al., 2020; Yumbul et al., 2010). The foundation of attachment-anxiety is generally believed to be a negative internal working model of the self as having an inability to respond to threat autonomously and fearing abandonment and inconsistency (Clark et al., 2020; Lin et al., 2020). Consequently, the present results suggest that when attachment-anxiety is activated, uncertainty may be assimilated into the aforementioned internal working model and perceived as indicative of threat (Wright et al., 2017). Given that caregivers that perpetrate CAN typically fail to guide a child’s emotion regulation in times of distress, which then acts to prevent the child from engaging with uncertainty and mastering the associated distress (Sanchez et al., 2016), the present finding suggests intolerance of uncertainty is a likely consequence of this (Wright et al., 2017). In relation to OCPT, the observed findings may be explained by attachment-anxiety inducing intolerance of uncertainty, which in turn, may lead to greater use of OCPT behaviours as a dysfunctional overcompensation to relieve affective distress. The latter paradoxically prevents disconfirmation of both attachment-related and ambiguity-related fears perpetuating OCPT psychopathology (Clark et al., 2020).

The finding above also points to future directions for research. When confronted with uncertainty and the associated distress, individuals with high intolerance of uncertainty attempt to instil a subjective perception of certainty by engaging safety behaviours (defined earlier; Clark et al., 2020). Interestingly, the behavioural manifestations of OCPT reflect efforts to exert excessive external (e.g., delegating reluctancy, interpersonal difficulties, organisation preoccupation) and internal overcontrol (e.g., perfectionism, moral scrupulousness, overconscientious). Thus, OCPT symptomology may be safety behaviours evolved as a maladaptive compensatory response to ambiguity (Clark et al., 2020; Wheaton & Ward, 2020). This presents an avenue for future research to implement an exposure paradigm (i.e., exposure to uncertainty) to investigate whether OCPT behavioural performance exerts a negative effect on distress reduction, just as experimental demonstrations have previously found safety behaviours to do (Sloan & Telch, 2002).

### Hypothesis 2

Our second hypothesis, that metacognition and attachment-anxiety would significantly, positively serially mediate between child emotional abuse and OCPT controlling for depression, was not supported. Nevertheless, although metacognition and attachment-anxiety were not found to have a serial mediation effect, the results demonstrated that metacognition had a sole mediation effect. In other words, higher child emotional abuse frequency predicted greater dysfunctional metacognition, which, in turn, predicted greater OCPT. Again, although causal conclusions cannot be inferred due to our study’s cross-sectional design, these preliminary results are partially consistent with the proposed theoretical model (Hayes, 2018; Salthouse, 2011) whereby an indirect effect occurs via a modelled single mediation path rather than the serial mediation path (see Figs. 5 and 6). This preliminary finding also extends prior cross-sectional and prospective research reporting a positive association between child emotional abuse and metacognition (Myers & Wells, 2015; Raes & Hermans, 2008; Spasojević & Alloy, 2002), and between metacognition and OCPT/obsessive–compulsive symptoms (García-Villamisar & Dattilo, 2015; Myers et al., 2009; Sica et al., 2007).

On the face of it, the finding concerning single mediation contradicts Gray and Boag’s (in press) finding that metacognition did not mediate the CAN–OCPT relationship. However, Gray and Boag (in press) noted that the specific type of CAN might be an important factor given some previous evidence (Myers & Wells, 2015; Raes & Hermans, 2008). Our study tested this, and the present finding indicates that metacognition likely exerts its influence in the child *emotional* abuse***–***OCPT relationship, rather than CAN generally. That is, our study suggests that dysfunctional metacognition is likely one mechanism that can help account for the development of OCPT following exposure to emotional abuse in childhood.

In the context of the S-REF model (discussed earlier; Wells, 2000), repeated exposure to the controlling, intimidating, isolating, and critical caregiving characteristic of child emotional abuse likely disrupts adaptive metacognitive formation, which then activates dysfunctional positive and negative metacognitive beliefs (defined earlier; Myers & Wells, 2015). For example, a child may develop a positive belief that worry and threat monitoring must be used in order to be prepared for future danger and distress (Myers & Wells, 2015). A child may also hold a negative belief that constant intrusive thoughts about abuse means the thoughts must be true (e.g., an intrusion of being called useless by a caregiver means “I *must* be useless”) and they are to blame for the abuse (e.g., “If I was not useless, my caregiver would not have abused me”; Wells & Sembi, 2004). Additionally, dysfunctional metacognitions direct cognitive focusing on distress-congruent information which prevents successful navigation of affective distress (Myers & Wells, 2015; Wells & Sembi, 2004). Thus, the present finding suggests that metacognition may increase OCPT as an ineffective behavioural self-regulation pattern, perpetuating OCPT psychopathology.

The finding above also points to future directions for research. According to the S-REF theory, dysfunctional metacognitive beliefs trigger the cognitive attentional syndrome (discussed earlier) which is locked onto worry and threat and prevents the return of cognition to normal threat-free processing (Wells & Sembi, 2004). This cognitive attentional syndrome reinforces metacognitive beliefs and saturates low-level automatic processing (Myers & Wells, 2015; Scarpa et al., 2009). Thus, the cognitive attentional syndrome may heighten psychopathology by preventing the implementation of alternate thoughts and the disconfirmation of feared outcomes (Myers & Wells, 2015; Wells & Sembi, 2004). Consequently, future research should replicate this study incorporating the Cognitive-Attentional Syndrome Questionnaire (CAS-1; Kowalski & Dragan, 2019) to examine a theoretical model whereby the child emotional abuse–OCPT relationship involves an indirect serial mediation mechanism of metacognition and cognitive attentional syndrome in a sequential manner.

As discussed above, the current finding suggests that child *emotional* abuse is a particular precipitating factor of dysfunctional metacognition and subsequent OCPT. Following a traumatic childhood event, adaptive emotion self-regulation can still develop if the associated distress is successfully navigated with scaffolding from an emotionally responsive caregiver (Myers & Wells, 2015). However, past research reports that emotionally abusive caregivers typically have neither the ability to scaffold nor their own capacity for emotion regulation (Lavi et al., 2019; Morris et al., 2007). Thus, child emotional abuse may have a particularly strong influence on emotional regulation deficits in maltreated children in comparison to the other CAN types. Moreover, ineffective emotion regulation is conceptualised as a component of the cognitive attentional syndrome that metacognitive beliefs are reinforced by and lead to psychopathology through (Mazloom et al., 2016; Wells, 2000). This presents an avenue for future research to investigate metacognition (or domains of it) and difficulties in emotion regulation (or domains of it) as a bidirectional relationship that mediates the child emotional abuse–OCPT link. Additionally, future research would benefit from examining the strength of each CAN type in predicting difficulties in emotion regulation in an OCPT psychopathology sample.

There are, however, several considerations when addressing the finding that metacognition and attachment-anxiety did not appear to have a serial mediation effect. This may be due to our examination of metacognition as a general class. Myers and Wells’ (2015) exploratory work examined the mediation effect of just one of the five metacognitive domains relevant to psychopathology, suggesting that the negative beliefs about worry domain mediated between child emotional abuse and attachment-anxiety. Thus, the development of attachment-anxiety following exposure to *emotional* abuse in childhood may be particular to the activation of only a certain metacognitive domain/s. This presents an avenue for future work to explore the current hypothesised serial mediation with each of the five metacognitive domains individually. Our present findings also suggest that for attachment-anxiety to have an indirect effect in the CAN–OCPT relationship, the subsequent influence of heightened intolerance of uncertainty is required. Thus, we extrapolate a hypothesised conceptual model whereby the child emotional abuse–OCPT association may involve a serial-mediation of metacognition or particular metacognitive domains, attachment-anxiety, and intolerance of uncertainty in a sequential manner.

### Limitations

This study’s findings should, however, be considered in light of several limitations. Firstly, our study was cross-sectional, a design which limits definitive conclusions regarding causal and temporal relationships (Hayes, 2018; Maxwell & Cole, 2007). However, use of a cross-sectional design to examine mediation is recognised as an appropriate initial step to evaluate proposed theoretical mechanisms (Hayes, 2018; Salthouse, 2011; Wright et al., 2017). Consistent with this methodological practice, our study employed cross-sectional quasi-longitudinal mediation and found preliminary results that are consistent with the theorised mechanisms. Thus, the validity of our proposed serial mediation models should be further examined with future quasi-experimental or prospective research. On the other hand, as our study used a non-clinical undergraduate convenience sample, results may not generalise to clinical samples. However, both the FFOCI-SF (Hall-Jones et al., 2021) and SNAP-2 (Clark, 2014) have been found to generalise to clinical groups. Nevertheless, a priority of future research will be to validate the proposed theoretical mechanisms in a clinical sample. Similarly, the over-representation of female, young, and college-educated populations within our sample may reduce the generalisability of our findings to other, less homogenous populations. Moreover, while the present sample was only 49% Caucasian, the other ethnicities were under-represented (e.g., 0.7% Black). Concerning gender, however, OCPD traits appear to occur equally across the genders (APA, 2022), and child abuse and neglect as measured by the CATS has displayed little-to-no differences between genders (Sanders & Becker-Lausen, 1995). Given that the present sample found comparable results on the CATS as seen with previous samples, we believe that our findings are broadly generalisable. Nevertheless, future research should replicate the present study in an education-level, gender, and ethnically diverse sample(s) and examine the stability of our findings throughout the lifespan. Finally, retrospective self-reporting of CAN may be vulnerable to reporting refusal, over-reporting, or under-reporting due to recall biases, social desirability, denial, or symptom justification. However, reviews suggest retrospective self-reporting of early victimisation experiences is ethical and appropriate, has little recall bias, and is accurate and reliable (Hardt & Rutter, 2004).

### Future Research and Implications

Notwithstanding the preceding limitations, there are future research implications based on our findings. As mentioned previously, our inability to find a serial mediation of metacognition and attachment-anxiety presents a particular avenue of inquiry. Specifically, the question of which of the five metacognitive domain/s, if any, may form a serial mediation followed by attachment-anxiety in the child emotional abuse–OCPT relationship requires further exploration. Additionally, given the intricacies of the sequela of metacognition (e.g., Generalised Anxiety Disorder; Posttraumatic Stress Disorder), the development of dysfunctional metacognition following child emotional abuse is likely not sufficient for explaining OCPT (Sun et al., 2017). Thus, future research is needed to identify other likely untested mediators and mechanisms linking metacognition to OCPT following child emotional abuse. As mentioned above, such factors may include cognitive attentional syndrome as a second mediator or emotion regulation difficulties as a bidirectional relationship with metacognition. On a related note, further research could extend our proposed theoretical mechanism and investigate whether the sequence of metacognition (or particular domains of it), attachment-anxiety, and intolerance of uncertainty form an indirect chain between child emotional abuse and OCPT. Lastly, some individuals exposed to CAN do not develop adult OCPT and alternatively evidence positive developmental trajectories that enable adaptive functioning. Consequently, there are likely resilience factors in the CAN–OCPT link that function to buffer against the adverse sequela of abuse and neglect (Kwok et al., 2019; Valikhani et al., 2022). Thus, future research examining potential resilience factors that may represent additional mediators or mechanisms in the CAN–OCPT relationship is needed.

Our findings contribute preliminary evidence for mechanisms that may contribute to accounting for when CAN leads to OCPT, which has several potential clinical implications. The findings suggest the need for clinicians to assess patients presenting with OCPT for both attachment-anxiety and heightened intolerance of uncertainty, and for dysfunctional metacognition when there is a history of CAN and/or child emotional abuse respectively. This may aid clinicians to understand symptoms as potential regulators of a particular distress feeling(s) and to implement personalised clinical management on the basis of the patients experience of distress (Bach & Simonsen, 2021). Current treatment for obsessive–compulsive personality pathology employs cognitive therapy or cognitive-behavioural therapy (Diedrich & Voderholzer, 2015). Our findings suggest that it may be beneficial to incorporate into these existing treatment protocols a psychotherapeutic approach targeting both attachment-anxiety and intolerance of uncertainty. Our findings also suggest that metacognitive therapy, an effective and appropriate treatment that challenges and modifies metacognitions and increases cognitive flexibility and control (McEvoy, 2019), may be a new treatment protocol for reducing OCPT in patients with a history of child emotional abuse. More broadly, implementing clinical management for obsessive–compulsive personality pathology based on matching therapy to the relevant antecedent factors may help inform best use of treatment resources. This may further help expedite patient time in therapy and assist psychologists’ currently limited work-load capacity for new patients (i.e., 40% of psychologists have a waitlist; Bach & Simonsen, 2021; Stamm et al., 2023).

## Conclusion

This study was the first to investigate whether attachment-anxiety, intolerance of uncertainty, and metacognition form mechanisms that may help contribute to explaining why child abuse and neglect sometimes leads to OCPT and not at other times. The results provide preliminary evidence that attachment-anxiety and intolerance of uncertainty are a sequential indirect mechanism in the link between CAN and OCPT. The results further indicate that serial mediation is not occurring for the sequence of metacognition as a general class and attachment-anxiety in the child emotional abuse-OCPT link. In spite of the foregoing, the results provide preliminary evidence that metacognition has a mediation effect that may help contribute to accounting for why child emotional abuse sometimes lead to OCPT and not at other times. While future longitudinal research is required to elucidate causality, attachment-anxiety, intolerance of uncertainty, and metacognition all present promising targets for both the assessment and treatment of obsessive–compulsive personality pathology.

## Data Availability

The lead author has full access to the data reported in the manuscript. De-identified data will be made available upon reasonable request for the purposes of checking from the corresponding author. The data that support the findings of this study are stored in the Macquarie University Research Data Repository (based on Figshare for Institutions) at 10.25949/24313063.v1.

## Footnotes

1 Excluded participant demographics. Age: 18 (*n* = 5), 19 (*n* = 2), 21 (*n* = 1), and 22 (*n* = 1). Gender: male (*n* = 3) and female (*n* = 6). Ethnicity: Caucasian (*n* = 3), South Asian (*n* = 2), Asian (*n* = 1), South Indian (*n* = 1), and Afghan (*n* = 1).

## Declarations

### Author Agreement

All authors have seen and approved the final version of the manuscript being submitted. The article is our own original work, has not been published previously, and is not under consideration for publication elsewhere.
